# Micropuncture and granular hydrogel scaffolds to surgically bioengineer a perfusable and stably patterned microvasculature

**DOI:** 10.1007/s10456-025-10003-x

**Published:** 2025-09-10

**Authors:** Jessica C. El-Mallah, Zaman Ataie, Summer N. Horchler, Mary E. Landmesser, Mohammad Hossein Asgardoon, Olivia Waldron, Arian Jaberi, Alexander Kedzierski, Mingjie Sun, Amir Sheikhi, Dino J. Ravnic

**Affiliations:** 1 Division of Plastic Surgery, Department of Surgery, Penn State Health Milton S. Hershey Medical Center, Hershey, PA 17033 USA; 2 Department of Chemical Engineering, The Pennsylvania State University, University Park, PA 16802 USA; 3 Irvin Zubar Plastic Surgery Research Laboratory, College of Medicine, The Pennsylvania State University, Hershey, PA 17033 USA; 4 Department of Biomedical Engineering, The Pennsylvania State University, University Park, PA 16802 USA; 5 Huck Institutes of the Life Sciences, The Pennsylvania State University, University Park, PA 16802 USA; 6 Department of Chemistry, The Pennsylvania State University, University Park, PA 16802 USA; 7 Department of Neurosurgery, College of Medicine, The Pennsylvania State University, Hershey, PA 17033 USA

**Keywords:** Microsurgery, Micropuncture, Granular hydrogel, Scaffold, Biomaterial, Vascularization

## Abstract

Vascularization of implanted biomaterials is critical to reconstructive surgery and tissue engineering. Ultimately, the goal is to promote a rapidly perfusable hierarchical microvasculature that persists with time and can meet underlying tissue needs. We have previously shown that using a microsurgical technique, termed micropuncture (MP), in combination with porous granular hydrogel scaffolds (GHS) fabricated via interlinking hydrogel microparticles (microgels) results in a rapidly perfusable patterned microvasculature. However, whether this engineered microvasculature remains stable at longer time points remains unknown. Here, we combine MP with GHS and compare overall microvascular architecture and phenotype along with the evolving cellular landscape over a 28 day period. We demonstrate perfusable patterned microvascular stability in our MP + GHS model that occurs alongside a sustained rise in endothelial cell and macrophage recruitment. Specifically, MP yields a significant rise in M2 macrophages between the 7 and 28 day time points, suggesting ongoing microvascular remodeling, even in the presence of early pericyte stabilization. With time, the GHS microvasculature acquires a relatively equivalent arterial and venous morphology, as assessed through Ephrin-B2 and EphB4 quantification. Finally, angiography at 28 days shows that MP + GHS is associated with more perfusable microvascular loops when compared with MP + Bulk (nonporous) scaffolds. Hence, our surgically bioengineered microvasculature offers a unique opportunity to sustainably and precisely control biomaterial vascularization and ultimately advance the fields of reconstructive surgery and tissue engineering.

## Introduction

Vascularization is paramount to clinical care across multiple entities. For reconstructive surgeons, the vascularization of biomaterials, such as collagen scaffolds, holds tremendous clinical significance. Biomaterial vascularization is also one of the most significant hurdles in tissue engineering and is a critical bottleneck to clinical translation. The challenge lies in promoting a rapidly perfusable microvasculature that can morphologically respond to underlying physiologic needs, matching form with function [1–3].

Native microvascular remodeling is a dynamic process, as seen during the immense growth phase occurring after birth [4–6]. Postnatally, the microvasculature undergoes continual development and expansion, primarily via angiogenesis, to meet metabolic demands. Remodeling is driven by changes in the surrounding cellular landscape, mediated through alterations in growth factor signaling. This leads to initial endothelial cell (EC) proliferation and eventual microvascular stabilization that acquires both arterial and venous characteristics [7–9]. Pericytes are critical to final stabilization. Macrophages, also abundant during this process, function in an inflammatory capacity early on before exerting tissue remodeling effects that are largely controlled by specific sub-phenotypes. Consequentially, crosstalk between ECs, pericytes, and macrophages is critical to normal microvascular development and maturation, as seen during wound repair. However, in many instances the patient’s wound precludes normal healing and necessitates reconstructive surgery [10–13].

Comparable examples of microvascular remodeling are seen by plastic surgeons who use scaffold biomaterials for tissue reconstruction [14, 15]. Ideally, effective biomaterial vascularization should occur rapidly, mimicking natural processes, with the supporting microvasculature effectively evolving in density, diameter, architecture, and complexity to match form with function —a cornerstone principle of reconstructive surgery.

However, controlling microvascular development that satisfies reconstructive needs poses complex challenges. Research has shown that vascularization strategies, such as the use of growth factors or pre-seeded scaffolds, are only marginally successful [16–19]. Unfortunately, these microvasculatures are not rapidly perfusable and lack a definable architecture with long-term stability. Ultimately, microvascular architecture sets the foundation for final tissue morphology. An optimal solution would both accelerate angiogenesis and provide the correct environment for precisely controlled scaffold microvascular development and maturation.

We recently described a microsurgical approach, termed micropuncture (MP), in which a targeted macro-blood vessel wall is perforated at specific intervals to provide an immediate route for inflammatory and EC extravasation, thereby accelerating microvascular outgrowths. When used in the presence of an adjacently placed hydrogel scaffold, the biomaterial is rapidly vascularized [20, 21], albeit with a random architecture. It also appears that the MP-induced randomly oriented microvasculature persists for at least one month [20]. Rapid vascularization is critical to biomaterial success in reconstructive surgery and for the development of new tissue-engineered solutions. However, true tissue building also requires an appropriately paired biomaterial that can ultimately match microvascular form with function.

Ideal materials are designed to mimic the biomechanical structure of extracellular matrices (ECMs) and facilitate host tissue integration by promoting cellular infiltration and angiogenesis. The major hurdle to complex tissue replacement is achieving a precisely patterned microvasculature that can carry oxygen, remove waste, and deliver cells uniformly in response to underlying needs. To overcome these challenges, void spaces within hydrogels have been engineered to promote and guide neovascularization. Engineering interconnected pores facilitates the cellular migration, proliferation, and nutrient exchange necessary for microvascular development. Granular hydrogel scaffolds (GHS) which consist of assembled hydrogel microparticles (microgels) enable the formation of interconnected pores in a controlled manner to support cell infiltration, vascularization, and tissue regeneration [22–24]. GHS have been investigated for a variety of regenerative applications, including skin wound healing and cardiac regeneration following infarction [25–28].

In our recent collaborative work, we developed a synergistic MP + GHS platform to rapidly promote and pattern microvasculature [29]. We have fabricated gelatin methacryloyl (GelMA) GHS with precisely defined void spaces through microgel photo-assembly [24, 30–32]. After performing MP in the rat femoral vasculature (artery and vein), GelMA GHS were adjacently implanted. After 1 week, the implanted scaffolds had a patterned microvasculature, with the intercapillary distances regulated by microgel size, and vascular density substantially augmented by MP [29]; however, it remains unclear if the MP + GHS-induced microvascular architecture persists with time. It may be rational to speculate that as the inflammatory effects of MP subside, so will the induced scaffold microvasculature. In this study we compare microvascular and cellular characteristics following 7 versus 28 implantation days with the hypothesis that MP + GHS results in long-term perfusable microvascular stability and architecture.

## Materials and methods

### Materials

Gelatin type A from porcine skin (≈ 300 g Bloom), methacrylic anhydride (MAA, containing 2000 ppm topanol A as an inhibitor, 94% purity), Dulbecco’s phosphate-buffered saline (DPBS), lithium phenyl-2,4,6-trimethylbenzoylphosphinate (LAP), and trichloro(1H,1H,2H,2H-perfluorooctyl)silane (F-silane) were purchased from Sigma, MA, USA. Vacuum filtration (pore size = 0.20 µm) systems were purchased from VWR, PA, USA. Ultrapure (Milli-Q) water (electrical resistivity ≈ 18 MΩ cm at 25 °C) was generated using a purification system from the Millipore Corporation, MA, USA. Dialysis membranes with 12–14 kDa molecular weight cutoff were purchased from Spectrum Laboratories, NJ, USA. Syringes (5 mL) were purchased from Becton Dickinson (BD, NJ, USA). Novec 7500 Engineered Fluid was purchased from 3 M, MN, USA. Pico-Surf (5 vol% in Novec) was purchased from Cambridge, UK, and 1H,1H,2H,2H-perfluoro-1-octanol (PFO) was purchased from Alfa Aeser, MA, USA.

Hanks’ balanced salt solution (HBSS) and phosphate-buffered saline (PBS, 0.1 µm sterile filtered, 1X, without Ca, Mg, phenol red) were obtained from Genesee Scientific, CA, USA. Ethanol (absolute, anhydrous, 200 proof) was purchased from Greenfield Global, CT, USA, and isoflurane was provided by Piramel, PA, USA. Betadine antiseptic povidone–iodine solution was obtained from Purdue Products, CT, USA, and 100% ethyl alcohol was obtained from Pharmco, CT, USA. Xylene, 10% Formalin solution, and 1M sterile filtered D-( +)-glucose aqueous solution was obtained from Fisher Scientific, MA, USA, and 1,1′-dioctadecyl-3,3,3′,3′-tetramethylindocarbocyanine perchlorate (DIL dye) and 25% gluteraldehyde solution were obtained from MilliporeSigma, MA, USA. Absorbable suture was from Ethicon, NJ, USA, and ElaSkin skin glue was from ALEO BME, PA, USA. The drugs Carprofen and Euthasol were from Zoetis, NJ, USA, and Virbac, TX, USA, respectively.

The primary antibodies used in this study were i) polyclonal goat immunoglobulin G (IgG) CD31/PECAM-1 antibody from R&D systems, MN, USA, ii) polyclonal rabbit IgG EMR1 (F4/80) antibody from Invitrogen, MA, USA, iii) monoclonal mouse IgG CD163 (ED2) antibody from Santa Cruz Biotechnology, CA, USA, iv) monoclonal rabbit IgG CD86 (B7-2) antibody from Invitrogen, MA, USA, v) polyclonal goat IgG Ephrin B2 antibody from R&D systems, MN, USA, vi) monoclonal mouse IgG EphB4 (5G2F8) antibody from Santa Cruz Biotechnology, CA, USA, vii) polyclonal rabbit IgG NG2 antibody from Invitrogen, MA, USA, and viii) monoclonal rabbit IgG α-smooth muscle actin (17H19L35) antibody from Invitrogen, MA, USA. The following secondary antibodies from Invitrogen, MA, USA were used: i) polyclonal donkey IgG AlexaFluor 488 PLUS anti-mouse antibody, ii) polyclonal donkey IgG AlexaFluor 594 PLUS anti-rabbit antibody, iii) polyclonal donkey IgG Alexafluor 488 PLUS anti-rabbit antibody and iv) polyclonal donkey IgG AlexaFluor 594 PLUS anti-goat antibody. Slides were mounted with EMS glycerol mounting medium with DAPI and DABCO from Electron Microscopy Sciences, NJ, USA.

### Gelatin methacryloyl (GelMA) synthesis

GelMA was synthesized following our established protocols [33]. In brief, 20 g of gelatin was dissolved at 50 °C in 400 mL of DPBS under constant stirring at 200 rpm. To initiate the reaction, 16 mL of MAA was added dropwise to the mixture, which was maintained at 40 °C, and the reaction vessel was shielded against light by wrapping in aluminum foil. The reaction was quenched after 2 h by adding 400 mL of additional DPBS. The resulting solution underwent dialysis against ultrapure water for 10 days at 40 °C to eliminate any unreacted MAA and side products. The dialyzed solution was then sterile filtered and subsequently frozen at -80 °C. Finally, the frozen GelMA was lyophilized using a Labconco FreeZone 4.5L -84 °C Benchtop Freeze Dryer (Labconco Corporation, MO, USA) at 0.009 mbar (collector temperature ≈ -82.4 °C) to yield a white solid GelMA polymer.

### Microgel fabrication

The droplets were fabricated using step-emulsification microfluidic devices, followed by our published protocol [24, 33]. In short, a 10% w/v GelMA solution was prepared by dissolving GelMA polymer in a LAP solution (0.1% w/v in DPBS) at 40 °C. This solution was used as a dispersed (aqueous) phase in the droplet fabrication process. The continuous (oil) phase consisted of Novec Engineered Fluid and 2% v/v surfactant (Pico-Surf). The aqueous and oil phases were independently loaded in 5 mL syringes and introduced into the microfluidic device using syringe pumps (PHD 2000, Harvard Apparatus, MA, USA) to form GelMA droplets. The setup was maintained at around 40 °C using a space heater to prevent GelMA physical gel formation. The GelMA droplets were collected, shielded from light, and maintained at 4 °C overnight to physically crosslink and yield GelMA microgels.

### GHS fabrication

The oil and surfactant were removed from the physically crosslinked GelMA microgels using an equal volume of PFO (20% v/v in Novec Engineered Fluid). The mixture was vortexed briefly and then centrifuged at 300 × ***g*** for 15 s. After discarding the supernatant, a 0.1% w/v LAP in DPBS solution was added to the microgels at a 1:1 volume ratio. The microgel suspension underwent vortexing and centrifugation at 300 × ***g*** again for 15 s, and the excess solution was removed. The microgels (average diameter of 81 ± 4 µm) [29] were then packed via centrifugation at 3000 × ***g*** for 15 s and moved to a custom-made acrylic mold placed on a glass slide using a positive displacement pipette (Microman E M100E, Gilson, OH, USA). Finally, the microgels were photochemically crosslinked by light exposure (wavelength of 395–400 nm and intensity of 15 mW cm^−2^ for 1 min) to form GHS. The GHS void fraction was 22 ± 3%, and the median equivalent pore diameter was 20 ± 2 µm, as previously reported [29].

### Bulk hydrogel scaffold formation

GelMA bulk hydrogel scaffolds were fabricated via a two-step crosslinking method to replicate the GHS crosslinking. Briefly, lyophilized GelMA was dissolved in a 0.1% w/v LAP solution in DPBS to yield a 10% w/v polymer solution. The solution was maintained at 37 °C for 2 h to ensure complete GelMA polymer dissolution. To prevent premature gelation, pipetting was carried out using pre-warmed pipette tips (37 °C), and the solution was dispensed into laser-cut acrylic molds. These molds were placed in a humidity-controlled chamber at 4 °C overnight, enabling physical crosslinking through thermal gel formation. Following physical stabilization, photocrosslinking was conducted using a 395–400 nm light source at 15 mW cm⁻^2^ for 1 min, forming covalently crosslinked bulk hydrogel scaffolds.

### Micropuncture (MP) and GHS implantation

Precision MP was performed in rat femoral vessels (artery and vein). Animal surgery was compliant with the Institute Animal Care and Use Committee (IACUC; 47,941)-approved protocol at The Penn State Hershey Medical Center. Sprague–Dawley (SD) rats were used at around 12 weeks of age (Charles River, MA, USA) and an equal number of male and female animals were used for the surgeries to minimize gender differences. Rats were anesthetized with isoflurane, and surgical sites were shaved and prepped with a betadine solution. Incisions were made over the inner hindlimb for femoral vessel exposure. Circumferential femoral vessel dissection was conducted along a 15 mm vessel segment in both hindlimbs. Then, 15 MP were created at 1 mm intervals using a 60 μm-diameter needle along the length of exposed vessel in one hindlimb. No MP was performed in the contralateral hindlimb. GHS or bulk GelMA scaffolds were then placed directly over the exposed vessels, and the overlying soft tissue was closed over the scaffold. Buried absorbable suture was used to close the skin with ElaSkin skin glue applied on top. A single dose of subcutaneous Carprofen was given for pain control. Standard post-surgical care was provided for the rats, including placement in individual cages with a 12 h day/night light cycle and ad lib food and water. Animals were euthanized via intracardial injection of Euthasol sodium pentobarbital phenytoin solution at either 7 or 28 days, and the scaffolds were explanted for further analysis. Test groups included MP + GHS at day 7 (*n* = 12 hindlimbs), no MP + GHS at day 7 (*n* = 12 hindlimbs), MP + GHS at day 28 (*n* = 10 hindlimbs), no MP + GHS at day 28 (*n* = 10 hindlimbs), MP + Bulk GelMA hydrogel scaffold at day 28 (*n* = 10 hindlimbs) and no MP + Bulk GelMA hydrogel scaffold at day 28 (*n* = 10 hindlimbs).

### Immunofluorescence staining

Immunofluorescent staining was used to characterize cell infiltration into the scaffolds (*n* = 3 scaffolds per condition). Anti-CD31 (EC marker), anti-F4/80 (macrophage), anti-ephrinB2 (arterial EC), anti-EphB4 (venous EC), anti-CD86 (M1 macrophage), anti-CD163 (M2 macrophage), and anti-NG2 (pericyte) antibodies were used. Slides were prepared for staining by deparaffinizing and rehydrating the tissue by immersion in xylene three times for 5 min, in 100% ethanol two times for three min, then in 95% ethanol two times for two min each, and then in 70% ethanol once for two min, followed by three washes with deionized water for 3 min each. Secondary antibodies conjugated to AlexaFluor 488 or 594 were applied, and mounting medium with DAPI and DABCO was used to mount the samples. The EVOS FL Auto Imaging System (ThermoFisher Scientific, MA, USA) was used to obtain images. A minimum of 15 images at 20 × were used for the CD86, CD163, Ephrin B2, Eph B4, and NG2 groups, and 20 images at 10 × were used for CD31, DAPI and F4/80 quantification. Staining area was quantified using FIJI ImageJ software (1.53t, NIH, MD, USA) [34].

### Perfusability demonstration (angiogram)

On day 28, in situ scaffold (*n* = 3 per group) perfusion was assessed (MP + GHS, no MP + GHS, MP + Bulk GelMA hydrogel scaffold, and no MP + Bulk GelMA hydrogel scaffold) using a previously described fluorescence vessel painting technique [35]. Under general anesthesia, the descending aorta was cannulated with an olive-tipped cannula (Medtronic DLP 1.8″ internal mammary artery cannula; Dublin, Ireland). The inferior vena cava (IVC) was transected to permit fluid efflux. PBS at 37 °C was first used to flush the lower extremities clear of blood. The hindlimb vasculature was then intravascularly fixed with 40 mL of 2.5% v/v glutaraldehyde solution. Rodent tail and limb stiffness were used to confirm fixation. Following fixation, the hindlimbs were perfused with lipophilic carbocyanine dye. Briefly, the dye was prepared by first dissolving carbocyanine in ethanol to create a 6.42 mM stock solution. Just prior to the injection, the stock solution was diluted (1:50) with PBS containing glucose (200 mM) to a final concentration of 0.128 mM. Then, 40 mL of the DIL lipophilic tracer was injected, and adequacy of perfusion was confirmed by distal nail bed color transition to pink. Scaffolds and the traversing femoral vessels were then explanted *en bloc*. The explants were prepared as whole mounts and fixed in 10% w/v formalin for 24–48 h. Distilled water was used to rinse the specimens before mounting, and 20 images at 10 × magnification were captured from each group. The scaffold microvasculature was assessed with artificial intelligence (AI) to minimize bias using a customized and validated platform (MetaVi Labs, TX, USA) [20]. AI outputs included total tube length, mean vascular density, average vessel diameter, and vessel branching. As the AI platform had not been trained and validated to measure intercapillary distance (ICD), AI output images for this parameter were analyzed by three blinded observers.

### Statistical analyses

Data was statistically analyzed using the two-way analysis of variance (ANOVA), followed by Tukey’s post-hoc multiple comparison test. For the in situ perfusability assessments (Fig. 7), three-way ANOVA with two-level repeated measurements was performed, followed by Tukey’s post-hoc multiple comparison test. All analyses were performed using GraphPad Prism (version 9.5.0, GraphPad, MA, USA). Statistical significance was defined as *p* < 0.05, indicated as *p < 0.05, **p < 0.01, ***p < 0.001, ****p < 0.0001. non-significant (ns) differences: *p* ≥ 0.05.

## Results

**Fig. 1:**
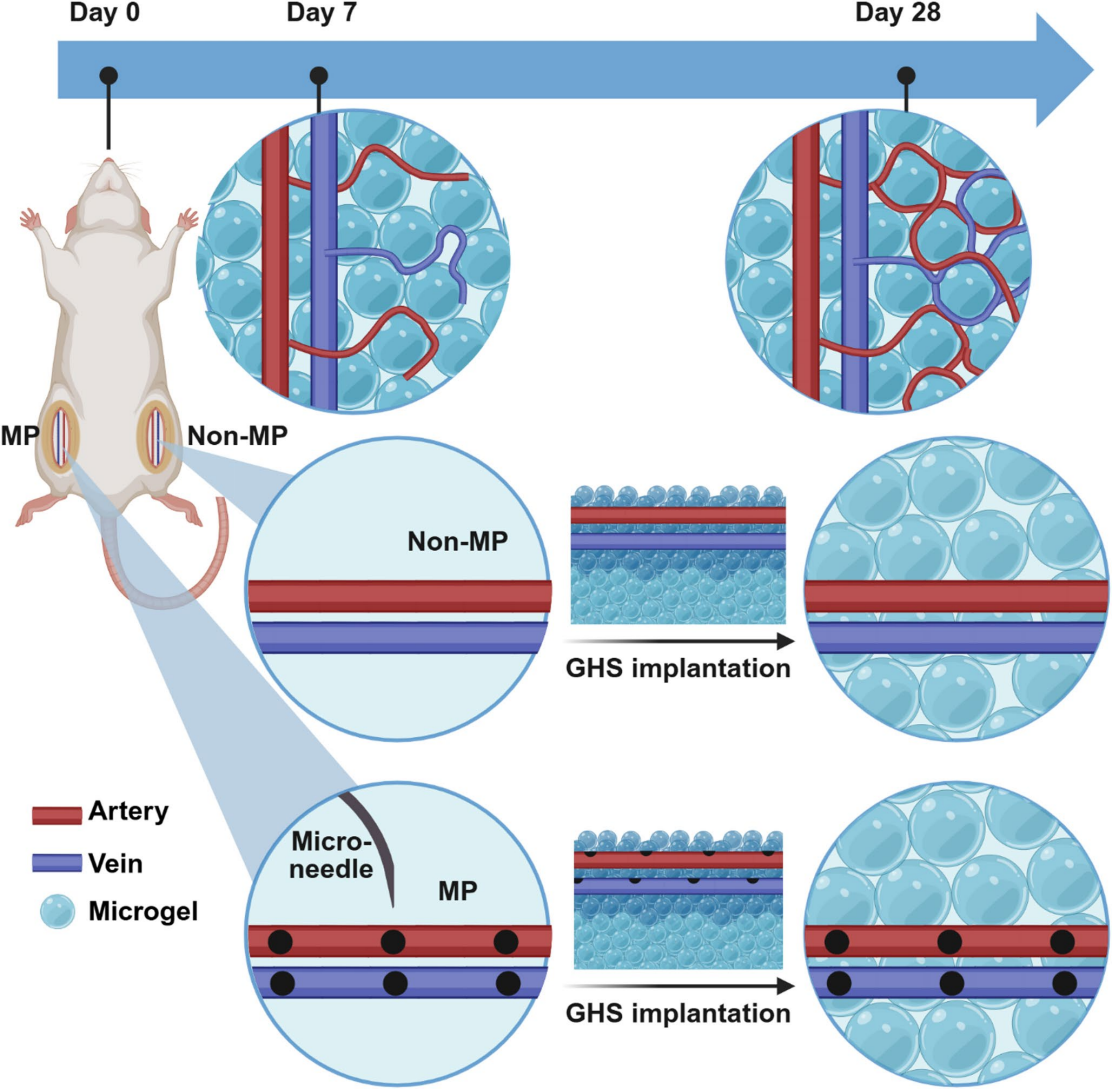
Experimental design and timeline for neovascularization in a rat hindlimb model

Figure 1 outlines the experimental design and timeline for scaffold vascularization in the rat hindlimb model. MP is performed into the femoral artery and vein of one hindlimb while the contralateral hindlimb serves as a non-MP internal control. Subsequently, scaffolds are implanted directly over the targeted macro-vessels, and the skin is closed. Scaffolds and traversing femoral vessels are explanted at either 7- or 28-days to analyze the microvasculature and/or cellular infiltration. Specific cell sub-populations such as ECs, pericytes, and macrophages are quantified.

On day 0, MP is performed on arteries and veins, followed by GHS implantation. In each rat, MP is applied to one hindlimb (MP group) and the other hindlimb is left untreated (non-MP control group). A hydrogel scaffold is then implanted in each hindlimb. Neovascularization and cell infiltration are assessed on days 7 or 28. Schematic not to scale.

**Fig. 2:**
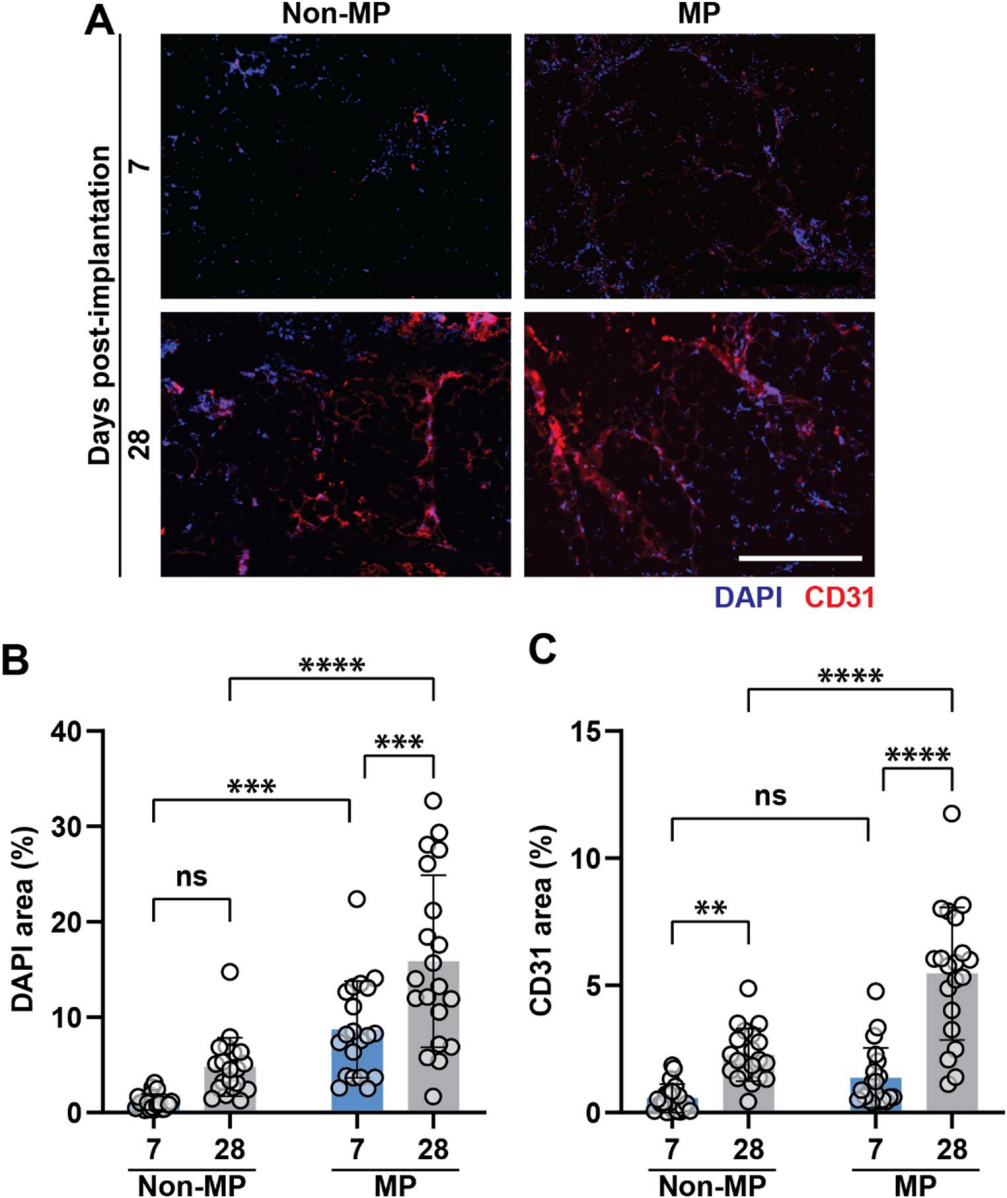
Cell infiltration in GHS. **A** DAPI (blue) and CD31 (red) staining showing nucleated and endothelial cell infiltration in GHS, respectively. The scale bar is 400 μm. **B** DAPI area quantification, showing that MP elicits significantly higher cell infiltration that increases over time. **C** CD31 area quantification showing MP augments EC infiltration into scaffolds on day 28 and increases with time. Two-way ANOVA is performed, followed by the Tukey’s multiple comparison test (ns is nonsignificant, *p* > 0.05; **p < 0.01; ***p < 0.001; ****p < 0.0001). MP increases scaffold microvascular development [20, 21, 29], but the underlying cell phenotypes have not yet been identified

Figure 2A shows DAPI staining to assess cell infiltration into the GHS. As quantified in Fig. 2B, there are significantly more nucleated cells within the scaffolds in the MP group compared to the non-MP controls after 7 days. This effect from MP is also observed at 28 days. Comparing day 7 versus day 28 cell infiltration, there is not a statistically significant increase in the non-MP control group; however, in the MP group, cell infiltration into the scaffold increases. Taken together, these data suggest that MP augments cell infiltration into GHS, and the effect is sustained over 28 days.

CD31 staining is used as a marker for EC infiltration into GHS. EC infiltration increases with time across both the non-MP and MP cohorts, suggesting that scaffold vascularization is ongoing within 28 days (Fig. 2C). Figure 2C shows that, although not significantly, MP increases EC infiltration at 7 days. Its effect is sustained, and by 28 days, there is a substantial increase of EC into GHS in the MP cohort. Again, this suggests that MP has a sustained effect in modulating the microenvironment and GHS vascularization within 28 days.

**Fig. 3:**
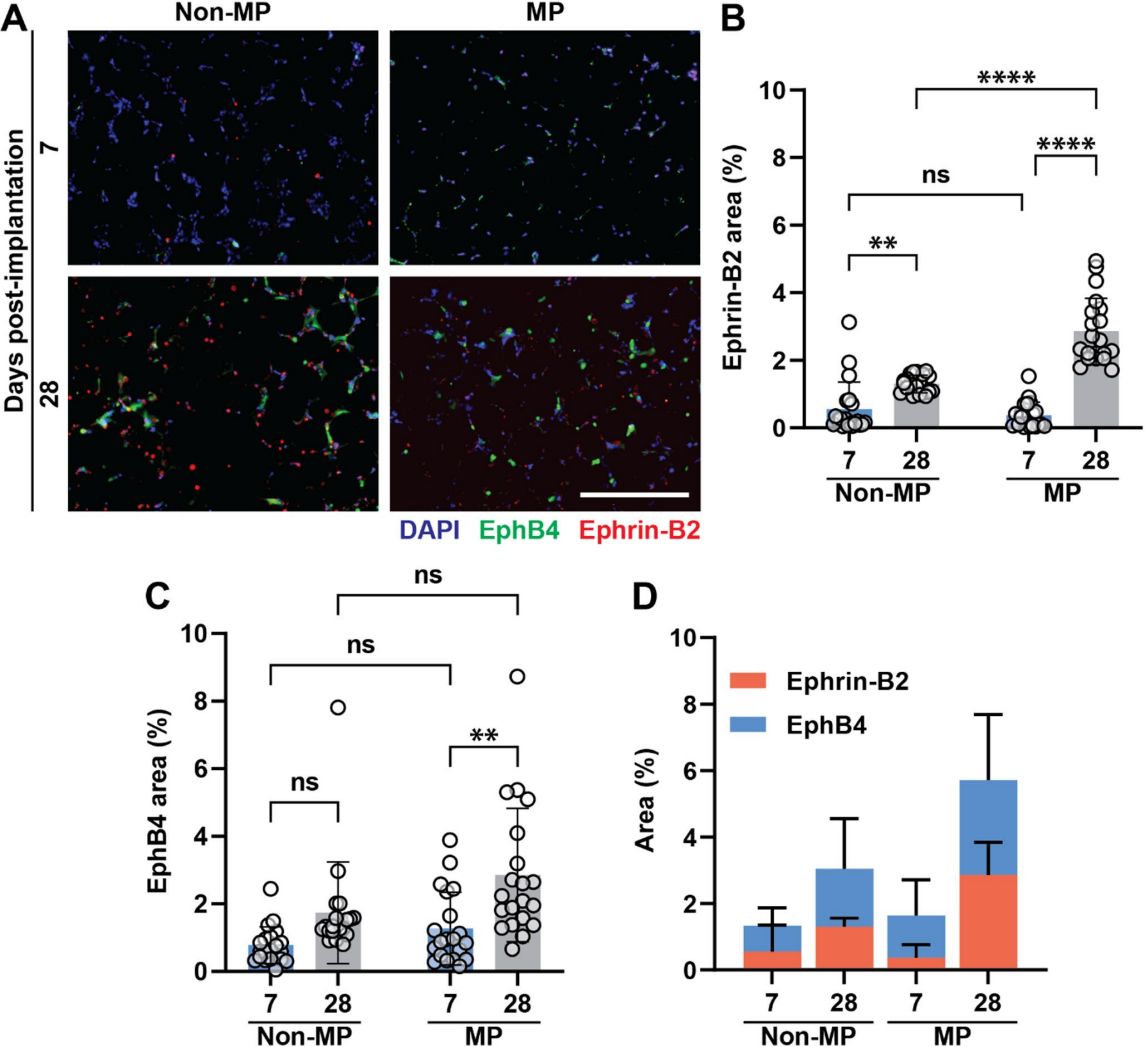
Arteriovenous differentiation. **A** Ephrin-B2 (arterial EC marker) and EphB4 (venous EC marker), staining at 7 and 28 days (DAPI as blue, Ephrin-B2 as red, EphB4 as green). The scale bar is 400 μm. **B** Quantification of Ephrin-B2 staining area. **C** Quantification of EphB4 staining area. **D** Comparison of Ephrin-B2 and EphB4 staining areas across experimental groups. Two-way ANOVA is performed, followed by the Tukey’s multiple comparison test (ns is nonsignificant, *p* > 0.05; **p < 0.01; and ****p < 0.0001)

In Fig. 3A, scaffolds are stained for Ephrin-B2 and EphB4, markers of arterial and venous EC phenotypes, respectively. Area quantification in Fig. 3B shows that Ephrin-B2 has a higher staining area at 28 days compared with 7 days both in the non-MP (control) and MP groups. This effect is much more profound with MP. EphB4, however, does not statistically change in the non-MP groups between days 7 and 28, though with the addition of MP there is significant expansion of EphB4 staining (Fig. 3C). Figure 3D further shows the area percentages of Ephrin-B2 to EphB4 in each experimental group. While there is no significant difference in Ephrin-B2 or EphB4 staining in the 7-day MP versus non-MP group, at the 28-day timepoint, there is significant expansion of EphB4 and even more so of Ephrin-B2. When considering all factors, MP appears to induce a scaffold microvasculature that is primarily venous in origin early on but with time develops equivalent arterial and venous components.

**Fig. 4:**
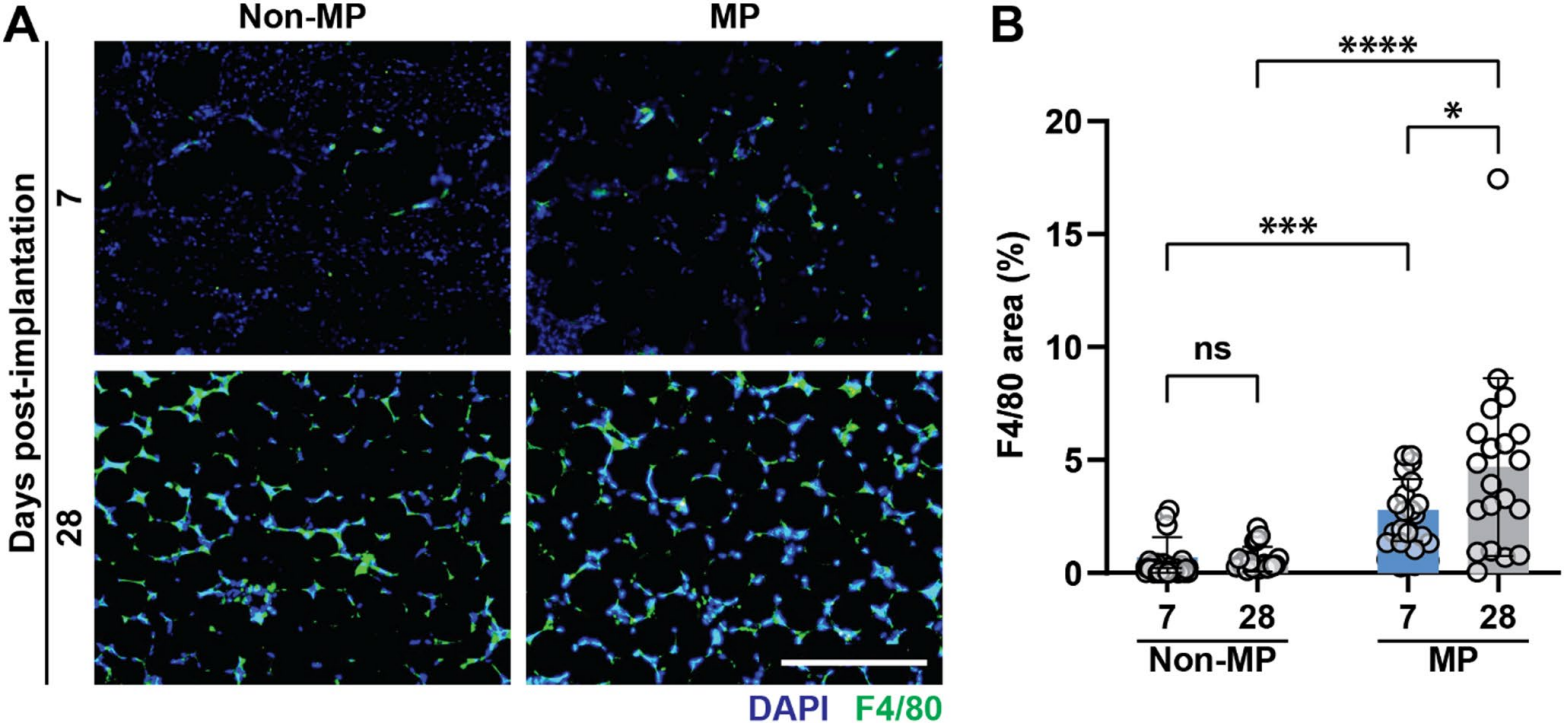
Macrophage recruitment. **A** F4/80 and DAPI staining in green and blue, respectively. The scale bar is 400 μm. **B** Quantification of F4/80 area, showing the effects of MP on macrophage recruitment into GHS over time. Two-way ANOVA is performed, followed by the Tukey’s multiple comparison test (ns is nonsignificant, *p* > 0.05; ****p < 0.0001)

It is noted that the increase observed in EC, irrespective of phenotype, does not fully account for all nucleated cells migrating into the scaffolds, as observed with DAPI staining. This likely indicates that there are multiple other cell types contributing to scaffold cellularization. Macrophages are known to play a crucial role in angiogenesis [36–38]. To explore their connection to the increased GHS vascularization after MP, scaffold macrophages are stained with F4/80 (green), as shown in Fig. 4A. Macrophages accumulate in the MP samples both at day 7 and 28, with the effects of MP being more pronounced at the longer time point (Fig. 4B**)**. This shows that MP promotes continued GHS inflammatory cell infiltration over time.

**Fig. 5:**
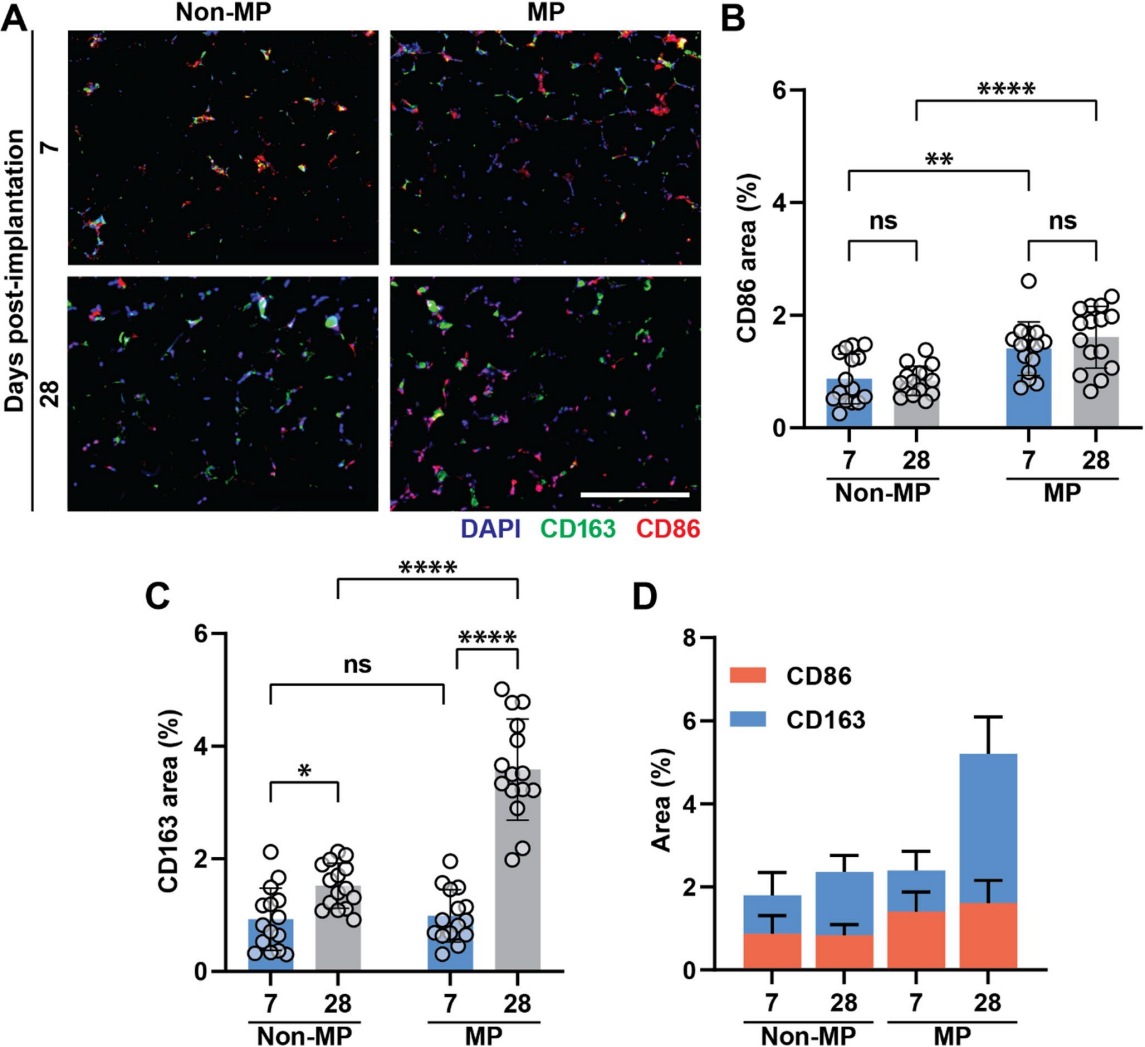
Macrophage polarization. **A** Staining for CD86, CD163, and DAPI in red, green, and blue, respectively. The scale bar is 400 μm. **B** CD86 expression is higher in MP samples compared with non-MP samples, with no significant differences observed between the 7-day and 28-day timepoints. **C** CD163 expression significantly increases at the 28-day timepoint in both non-MP and MP groups, with further enhancement observed in the MP. **D** Comparison of CD86 versus CD163 levels in each experimental group. Two-way ANOVA is performed, followed by the Tukey’s multiple comparison test (ns is nonsignificant; *p* > 0.05; *p < 0.05; **p < 0.01; ****p < 0.0001)

To further characterize the inflammatory response, specific subpopulations of macrophages are assessed at each time point, as shown in Fig. 5A. Cells within the scaffolds are stained for CD86 and CD163 markers of M1 and M2 macrophages, respectively, to delineate macrophage plasticity following MP. Specifically, CD86 and CD163 are quantified, as shown in Fig. 5B and C, respectively [39, 40]. Figure 5D presents the relative area of CD86 versus CD163 in each group. M1 macrophages are known to be pro-inflammatory, releasing cytokines to recruit additional inflammatory cells [12, 41]. Whereas M2 macrophages promote angiogenesis and are anti-inflammatory, promoting tissue repair and remodeling [12, 13]. Comparing 7- versus 28-days, only the M2 macrophage subpopulation increases at the longer time point, both in the non-MP and MP groups; however, MP is associated with a profound rise in M2 over time. This suggests that MP promotes ongoing tissue repair and remodeling that yields enhanced scaffold microvascular development.

**Fig. 6:**
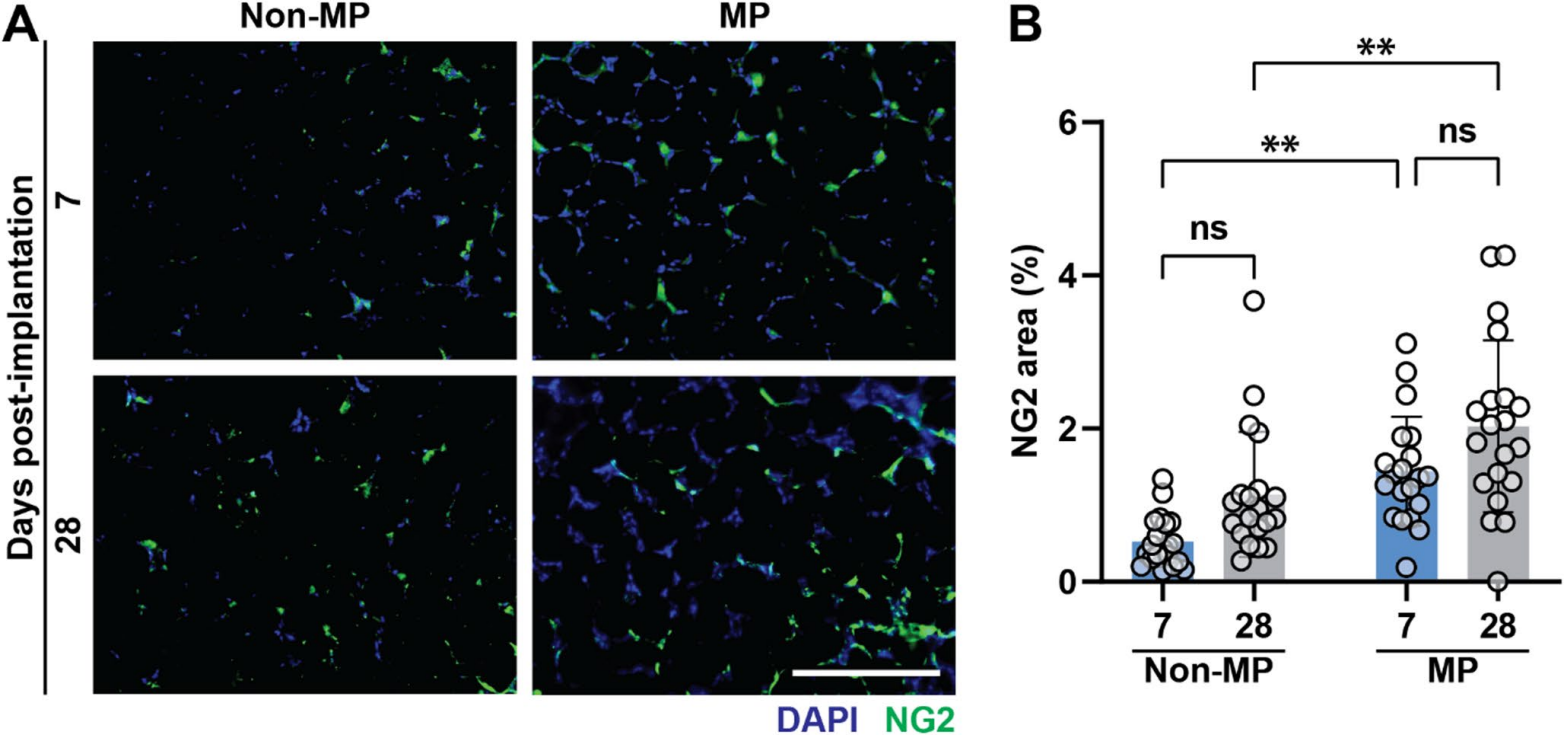
Pericyte infiltration. **A** NG2 staining (DAPI as blue, NG2 as green). The scale bar is 400 μm. **B** NG2 area quantification, showing that MP has a significant effect on NG2 expression both at 7- and 28-day timepoints. Two-way ANOVA is performed, followed by the Tukey’s multiple comparison test (ns is nonsignificant, *p* > 0.05; **p < 0.01)

Vascular support cells are integral to microvascular development and long-term stability. Pericytes are one such cell type [42–44]. Neural/glial antigen 2 (NG2), is a proteoglycan that serves as a pericyte marker [42–44]. To understand how MP affects pericyte recruitment, GHS were stained for NG2, as shown in Fig. 6A. Figure 6B shows that pericyte recruitment is higher in the MP samples at both time points, although there is no significant increase from day 7 to day 28. These data suggest that MP allows for the rapid influx of pericytes into the GHS, which may enhance early microvascular stabilization and promote sustained perfusability.

**Fig. 7:**
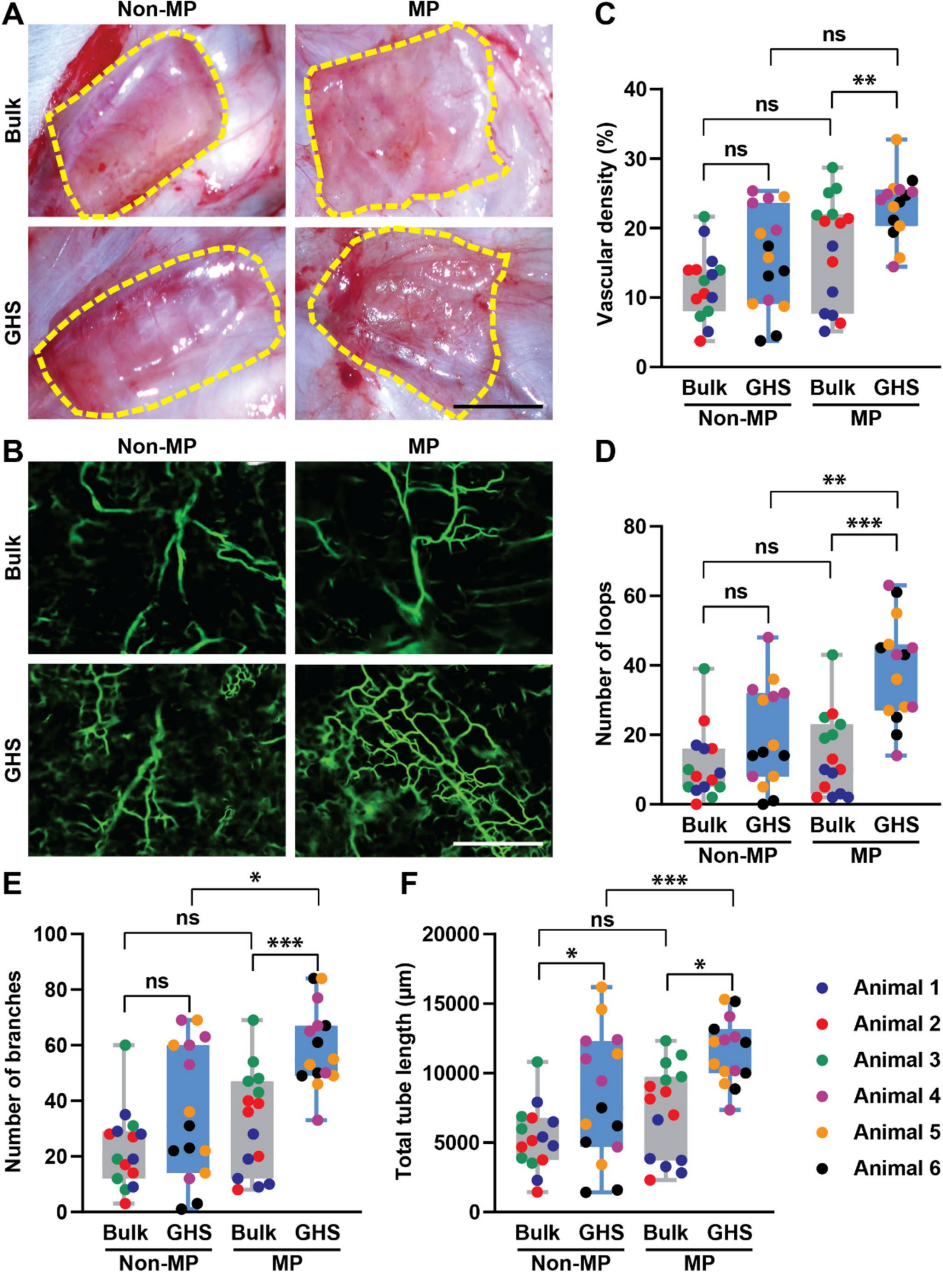
In vivo perfusion assessment of GHS and bulk hydrogel scaffolds with or without MP at day 28. **A** Gross images of implanted scaffolds 28 days after implantation. **B** Fluorescence angiograph images, showing significantly more perfusable and patterned microvascular in MP + GHS. The scale bar is 400 μm. AI-based quantification of **C** vascular density, **D** loop counts, **E** number of branches, and **F** total tube length in implanted scaffolds. A repeated-measures three-way ANOVA is performed, with each rat as a matching factor, followed by the Tukey’s multiple comparison test (ns is nonsignificant, *p* > 0.05; **p < 0.01)

Next, to quantify the degree of in vivo GHS perfusability, in situ angiograms are performed at 28 days with scaffold explants prepared as whole mount using a fluorescent vessel painting technique [35]. A validated AI platform was used for scaffold microvascular analysis. AI allows for the rapid and rigorous identification of perfused vessels, total scaffold vascular density quantification, and microvascular architecture delineation via counting vessel loops, branches, and lengths. To demonstrate the ability of GHS in guiding microvascular development, a GelMA bulk hydrogel scaffold, fabricated at the same GelMA concentration and crosslinking conditions as in the GHS, is used as a secondary control. Figure 7 shows the experimental groups at 28 days, comparing a bulk scaffold to GHS with or without MP. Figure 7A shows gross in situ images at the time of explantation. Although not quantified, there is a visible increase in the vascularity of the GHS + MP group compared with the other groups. Figure 7B shows angiograms from each group following intravascular dye perfusion and AI detection. GHS + MP group shows significantly greater microvascular density compared with the bulk + MP group (Fig. 7C). GHS + MP also has a greater number of loops and branches, undergoing enhanced vascularization, with a slight increase in tube length only noted in the GHS groups (Figs. 7D-F). This suggests that the increased vascular density resulted from vessels wrapping around the microgels within the GHS that is augmented by the MP effects. This appears different from bulk scaffolds in which new microvascular development is not patterned, as suggested by the absence of continuous loops.

Our initial studies evaluated GHS as compared to bulk scaffolds with or without MP at the 7- day time point [29]. We have excluded these findings here as they have been published [29]. While our initial results suggested that MP + GHS hastened the development of perfusable vessels as compared with bulk hydrogel non-MP control; our results outlined here suggest that the MP + GHS microvasculature continues to persist with time with a definable loop architecture while the bulk hydrogel scaffold microvasculature matures in a random fashion. This complex vascular network topology more closely resembles the native microvasculature of soft tissues. While this results in more loops and tortuous paths, such complexity is a hallmark of physiologic microvascular beds, where vessel redundancy and branching provide alternative perfusion routes and resilience against localized flow disruptions.

**Fig. 8:**
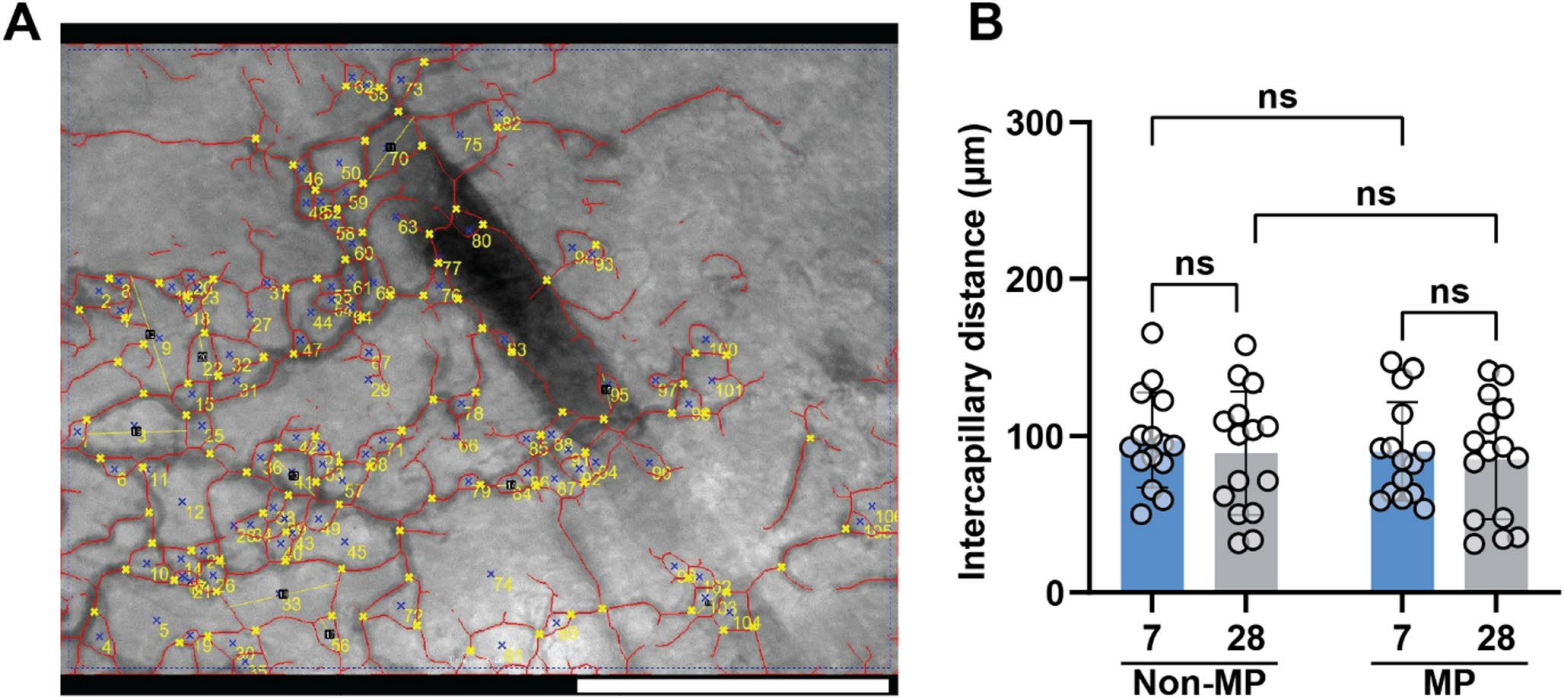
ICD measurement. **A** Images in which vessels are traced by AI (vessels in red), with loop counts shown by blue x. Blinded observers measured the loop diameter, as marked by yellow lines. The scale bar is 400 μm. **B** Quantification of ICD, conducted by observers. Two-way ANOVA is performed, followed by the Tukey’s multiple comparison test (ns is nonsignificant, *p* > 0.05)

Lastly, the effect of MP and time on intercapillary distance (ICD) is measured. The diameter between AI-traced vessel loops is measured by 3 blinded observers **(**Fig. 8A**)**. ICD is similar across both 7- and 28-day timepoints, as well as between MP and non-MP groups **(**Fig. 8B**)**. These data suggest that the microvasculature is patterned by GHS and corresponds to the engineered microgel diameter, which is ~ 80 µm in these studies. Additionally, based on these measurements, vascular loops established with GHS persist over time. Whether or not this is because the GHS themselves persist or degrade is unclear, though the loops remain stable.

## Discussion

Microvascular engineering is essential to advance both reconstructive surgery and tissue engineering. Here, we add to our existing bodies of work that a coordinated microsurgical and bioengineering approach can facilitate the building of a patterned microvasculature that is stably perfusable. This affords surgeons the ability to facilitate angiogenesis and biomaterial vascularization that can be customized depending on clinical needs.

MP appears to stimulate microvascular development by triggering a large amount of nucleated cell infiltration into the adjacently placed scaffold. This is facilitated by the porous nature of GHS, compared with bulk hydrogel counterparts. Over time the increased accumulation of ECs, pericytes, and M2 macrophages manifests as a perfusable patterned microvasculature that has both arterial and venous characteristics. Here we observed a significantly elevated level of microvascular development in our MP + GHS group. Considered alongside an elevated level of M2 macrophages, it is suggested that by the 28-day timepoint there has been a shift from an acute inflammatory response towards reparative microvascular remodeling.

It is important to note that persistent angiogenesis in biomaterial implants does not necessarily equate to ongoing “wound healing” in the traditional inflammatory sense. Rather, it often reflects the maturation and adaptation of the neovasculature to meet the metabolic demands of the evolving tissue environment. In our study, macrophage recruitment increased over time, particularly CD163 + (M2-like) macrophages, without a corresponding rise in CD86 + (M1-like) cells. This suggests a shift toward a reparative and tissue-remodeling milieu rather than persistent inflammation. Such macrophage polarization has been shown to support vessel maturation, stabilization, and extracellular matrix remodeling rather than purely driving an inflammatory healing response.

Regarding microvascular stabilization, while angiogenesis is initially rapid following implantation (as early as 72 h in micropuncture models), data from our group and others demonstrate that the neovasculature formed within biomaterial scaffolds can persist and remain functional over longer periods, including up to 28 days, without evidence of continued inflammation [20]. However, ultimate stabilization—meaning the transition from active angiogenesis to a quiescent, mature microvascular network—is likely scaffold- and context-dependent. The literature suggests that significant vessel pruning and remodeling typically occur after initial vessel ingrowth and perfusion, often beyond the 28-day mark [45–47]. Therefore, while our findings indicate active vascular remodeling between day 7 and day 28, we anticipate that stabilization would follow as the metabolic and structural demands of the new tissue plateau. In summary, while increased vascularity at day 28 suggests ongoing neovascular adaptation rather than persistent wound healing, both processes are indeed intertwined in the early phases post-implantation. The transition to stabilization likely depends on achieving a balance between vascular supply and tissue demand, and further timepoints beyond 28 days would be informative in fully characterizing this progression.

A variety of factors, including environmental influences, determine whether a vessel is arterial or venous in morphology [48, 49]. We hypothesize that both MP and GHS provide unique environmental cues influencing the arteriovenous phenotype of the scaffold microvasculature, potentially independent of the hypoxia typically known to initiate microvascular development [50]. This is especially relevant to biomaterial vascularization as acellular scaffolds lack the inherent ability to release hypoxia inducible factor (HIF). Alternatively, MP-induced changes in fluid shear stress may contribute to this process as shear stress has been shown to play a role in EC homeostasis and microvascular development [51]. Future studies will examine these in further detail to mechanistically identify how the MP + GHS interface modulates microvascular formation and phenotype. One notable limitation to our arteriovenous data presented above is that the immunofluorescence images depict labeling primarily at the single-cell level rather than continuous vessel segments. While increased EphrinB2 and EphB4 staining correlates with endothelial cell infiltration and vascular maturation, we cannot exclude the possibility of signal originating from other cell types such as macrophages, which have been demonstrated to express ephrin ligands in the literature though not in the context of tissue engineering or vascular development. [52–54].

Notable in this work is the rapidity by which MP triggers pericyte infiltration into GHS, possibly conferring earlier microvascular stability. Also, pericytes play a role in modulating EC proliferation and migration that ultimately affects final microvascular architecture. In the initial stages of sprouting angiogenesis, pericytes detach from the vessel basement membrane to increase vascular permeability, facilitating EC migration into surrounding tissues [55]. As vessels sprout, EC secrete growth factors to further recruit pericytes into the budding vessels [56]. Consequentially, EC-pericyte interplay is profoundly important to native microvascular development. This process is still poorly described with regards to biomaterial vascularization. Future studies will investigate how GHS influences pericyte biology, with and without MP.

Also, it should be noted that there exists a complex interplay between microvascular network architecture and perfusion efficiency with vascular density not automatically equating to improved perfusion under physiologic conditions [57, 58]. However, our data indicate that MP + GHS conditions promote a microvascular architecture that is more branched and interconnected, leading to more resemblance with vasculature existing in native soft tissue. Microvascular loops and tortuosity are a hallmark of physiologic microvascular beds, where vessel redundancy and branching provide alternative perfusion routes and resilience against localized flow disruptions. Each specific tissue type within native tissue possesses its own hallmark vascular tree characteristics. The increased branching observed in the MP + GHS group may reflect a biomimetic microvascular pattern that can support tissue viability under dynamic physiologic conditions. Most consequentially, our findings from both early (day 7) and later (day 28) timepoints demonstrate that the microvasculature formed within MP + GHS scaffolds remains perfused and functional. As shown in our perfusion analyses, including fluorescent vessel painting and histological evidence of RBC-filled lumens, the increased vascular complexity in the MP + GHS group does not impede perfusion. Instead, the presence of perfused lumens throughout the scaffold suggests that even the more tortuous microvascular architecture can effectively support blood flow. Regarding intercapillary distance, we believe the relatively constant measurements across groups reflect the spatial constraints imposed by microgel architecture. Because vessels tend to grow around individual migrogels of defined diameter, the distance between vascular loops remains constrained, regardless of overall vascular density or branching complexity. Thus, the stability of intercapillary distances across groups and timepoints underscores the spatial consistency imparted by microgel design rather than indicating an absence of biological differences between groups. Collectively, our data suggest that the patterned microcirculation induced by MP + GHS scaffolds yields a physiologically relevant vascular network.

There are several limitations to this study. Primarily, more information is needed regarding GHS stability. Based on our in vitro results, we hypothesize that the microgels will likely degrade over time; however, the in vivo time course and its impact on microvascular morphology remains unclear. It is plausible that as scaffolds degrade, microvascular morphology will adjust to the underlying need of new tissue ingrowth. Longer implantation timepoints are needed to elucidate this.

Additionally, as these scaffolds were not placed in an isolation chamber, it is highly probable that some of the observed vascularity originated from adjacent tissues and not only the femoral vessels. In future studies, we will evaluate these processes within an isolation chamber to obviate the effects of extrinsic vascularization. Nevertheless, it is notable that the cellular effects of MP appear propagated over time, leading to sustained, and most importantly, patterned microvasculature, guided by the GHS pore microarchitecture.

In summary, our study shows that MP used alongside GHS creates a unique cellular interface that synergistically promotes a rapidly perfusable and stably patterned microvasculature. Consequentially, our coordinated surgical bioengineering vascularization platform may hold promise for both conventional reconstructive surgery and new opportunities in tissue engineering and regenerative medicine.

## Data Availability

Data is provided within the manuscript and is available for review upon request.
